# Development and Validation of a 6-Gene Hypoxia-Related Prognostic Signature For Cholangiocarcinoma

**DOI:** 10.3389/fonc.2022.954366

**Published:** 2022-07-18

**Authors:** Qi Sun, Huxia Wang, Baoan Xiao, Dong Xue, Guanghui Wang

**Affiliations:** ^1^ Department of General Surgery, The First Affiliated Hospital of Xi’an Jiaotong University, Xi’an, China; ^2^ Mammary Department, Shaanxi Provincial Cancer Hospital, Xi’an, China

**Keywords:** cholangiocarcinoma, hypoxia status, prognostic model, immune microenvironment, PPFIA4

## Abstract

Cholangiocarcinoma (CHOL) is highly malignant and has a poor prognosis. This study is committed to creating a new prognostic model based on hypoxia related genes. Here, we established a novel tumor hypoxia-related prognostic model consisting of 6 hypoxia-related genes by univariate Cox regression and the least absolute shrinkage and selection operator (LASSO) algorithm to predict CHOL prognosis and then the risk score for each patient was calculated. The results showed that the patients with high-risk scores had poor prognosis compared with those with low-risk scores, which was verified as an independent predictor by multivariate analysis. The hypoxia-related prognostic model was validated in both TCGA and GEO cohorts and exhibited excellent performance in predicting overall survival in CHOL. The PPI results suggested that hypoxia-related genes involved in the model may play a central role in regulating the hypoxic state. In addition, the presence of IDH1 mutations in the high-risk group was high, and GSEA results showed that some metabolic pathways were upregulated, but immune response processes were generally downregulated. These factors may be potential reasons for the high-risk group with worse prognosis. The analysis of different immune regulation-related processes in the high- and low-risk groups revealed that the expression of genes related to immune checkpoints would show differences between these two groups. We further verified the expression of the oncogene PPFIA4 in the model, and found that compared with normal samples, CHOL patients were generally highly expressed, and the patients with high-expression of PPFIA4 had a poor prognosis. In summary, the present study may provide a valid prognostic model for bile duct cancer to inform better clinical management of patients.

## Introduction

Cholangiocarcinoma (CHOL) is a malignant and aggressive disease with a poor prognosis, the median survival less than 24 months (1). Depending on the location of the tumor, CHOL is divided into intrahepatic cholangiocarcinoma (iCCA) and extra-hepatic cholangiocarcinoma (eCCA), and eCCA is divided into perihilar cholangiocarcinoma (pCCA) and distal cholangiocarcinoma (dCCA) (2). The early symptoms of CHOL are insidious and not easily detected, and most patients are diagnosed at an advanced stage (2). For patients with advanced CHOL, treatment options are limited. The median overall survival (OS) of patients treated with standard chemotherapy regimens (gemcitabine and cisplatin) is still less than 1 year (3). Therefore, it is clinically important to find the relevant factors affecting the prognosis of patients with CHOL. The American Joint Committee on Cancer (AJCC) staging manual has become a benchmark for classifying cancer patients, determining prognosis, and determining the best treatment (4). However, it has been discovered that the TNM staging approach is insufficient for determining prognosis and does not account for cancer’s biological heterogeneity. There is significant variation in prognosis and treatment response even among patients with the same stage, and other factors such as age, performance status, and tumor site might affect patient survival, therefore it only provides limited information on clinical prognosis (5). As a result, developing reliable prognostic biomarkers is critical in order to improve clinical prognostic value. With the continuous improvement and development of high-throughput sequencing technology (6), it is possible to exploit potential molecular targets in the CHOL genome using bioinformatics technology. The development of promising molecular targets is essential to improve the application of targeted therapy for CHOL.

The tumor microenvironment is a complex and variable cellular microenvironment, which can not only provide the material conditions required for the growth and proliferation of tumor cells, but also change its composition through autocrine and paracrine secretion of tumor cells (7). It was reported that CHOL cells can construct their own favorable environment by secreting tumor-related regulatory mediators through the extracellular matrix and stromal cells, thus promoting the proliferation of CHOL cells and enhancing their resistance to treatment (8). Most solid tumors include hypoxia as a micro environmental trait, and developing tumors frequently live in hypoxic environments due to limited blood supply (9). The hypoxic conditions could enhance the angiogenesis, proliferation and invasive abilities of tumor cells (10, 11). Similarly, in CHOL studies, hypoxia has been found to increase the aggressiveness of CHOL (12). In iCCA studies, hypoxic environment was able to promote the progression of CHOL through upregulation of Rab1a (13). It has been found that in CHOL, hypoxia-induced Sonic Hedgehog signaling pathway regulates cancer stemness, epithelial-to-mesenchymal transition and invasion (14).

In the present study, the hypoxia-related genes for CHOL were mainly screened by GEO and TCGA-CHOL dataset. The prognostic signature characterized by hypoxia-related genes were filtered according to univariate and LASSO regression analysis, and the risk score of each samples were obtained. Subsequently, CHOL samples in TCGA were classified into high- and low-risk subgroups according to the risk score, and the differences between high- and low-risk subgroups were further explored, such as survival differences, clinical characteristics differences, enrichment differences, somatic cell mutation differences, and tumor immunomodulatory gene expression differences.

## Materials and Methods

### Pre-Processing and Data Collection

We utilized the R package TCGAbiolinks (15) to download the 36 CHOL RNA-seq data [log2(FPKM+1)] and relevant clinical information from the TCGA database (https://portal.gdc.cancer.gov/). The 36 samples with both available RNA-seq data and clinical information were used as the training set for hypoxia-related markers. The GSE107943 dataset was obtained from the GEO database (https://www.ncbi.nlm.nih.gov/geo/), which were used as the external validation set, containing available microarray expression data and clinical data of bile duct cancer samples. The microarray data preprocessing process was as follows: remove null probes and probes corresponding to multiple genes at the same time, and use the median expression value of these probes as the expression value of the gene when multiple probes correspond to the same gene.

### Prognostic Model Construction

The 200 potential hypoxia-related genes were retrieved from MSigDB’s HALLMARK HYPOXIA pathway (https://www.gsea-msigdb.org/gsea/index.jsp) (v7.4 version). All prognosis-associated hypoxia genes were screened using univariate Cox hazard analysis, with a p-value of 0.1 chosen as the cutoff. Utilizing the R package glmnet, the Lasso Cox regression model was built using all prognosis-related hypoxia genes, and candidate genes were penalized to exclude redundant components and reduce the risk of overfitting (16). A 10-fold cross-validation under the constraint of low bias is used to calculate the model’s penalty parameter (λ).

To determine the best prognosis-associated hypoxia signature, Lasso regression analysis was used, and the regression coefficients were calculated for each gene separately, and then the sample risk scores for multivariate Cox regression were calculated using the R package survminer (17), with the formula as follows:


$$\text{Risk Score}=\sum_{\text{i}=0}^{\text{n}}\text{βi}*\text{χi}$$

βi denotes the weight of each gene in Lasso Cox regression; χi denotes the expression level of each gene.

The CHOL patients were divided into high-risk and low-risk groups based on Risk score. Kaplan-Meier curves of survival differences between samples in the high-risk and low-risk groups were plotted, and significant differences in OS between groups were assessed based on log-rank tests.

### Correlation Analysis of Prognostic Models and Clinical Characteristics

The univariate and multivariate Cox hazard regression analysis were performed separately for risk scores and patient clinical characteristics (including age, gender, and stage) to determine whether the predictive power of the prognostic model was independent of clinical characteristics. Risk ratios (HR) and 95% confidence intervals were calculated for each candidate prognostic factor, with a p-value < 0.05 as the threshold of significance. In addition, differences in risk scores across age, sex, and stage subgroups were assessed based on the Wilcoxon rank-sum test.

### Gene Interactions Analysis

The expression correlation between genes in the prognostic model was assessed based on Pearson correlation analysis. In addition, the interactions of proteins encoding hypoxia-related genes were obtained based on the STRING database (http://www.string-db.org/), and protein-protein interactions (PPI) network of hypoxia-related genes were constructed, and the network graphs were visualized using Cytoscape (v3.9.1) to assess the interactions of the genes in the prognostic model.

### Functional Enrichment Analysis

Gene set enrichment analysis (GSEA) was used to identify biological processes that differed significantly between high- and low-risk group samples (18). We examined biological processes from the GO database and KEGG signaling pathways for significant variations between high- and low-risk group samples using the R tool ClusterProfiler (19). To screen the collection of substantially linked genes, a corrected p-value of less than 0.05 was employed as a criterion, and the p-value correction technique Benjamini Hochberg was applied.

### Somatic Cell Mutations

TCGA provided the tumor mutational data. The mutation genes were calculated using tumor mutational burden (TMB). The mutational data in both the high-risk and low-risk groups were analyzed using the R maftools package (20). P-values less than 0.05 were deemed statistically significant.

### Expression of Immune-Negative Regulatory Genes

The cancer immune cycle has emerged as the primary research framework for cancer immunotherapy. It describes a cyclical process in which the immune system eradicates cancer: Antigen Processing and Presentation (Category 1), Atimicrobials (Category 2), Chemokines (Category 3), Cytokine Recepors (Category 4), Cytokines (Category 5), Matrix remodeling (Category 6), and Nature Killing Cell Cytoxicity (Category 7) (21). In the low and high hypoxia risk groups, we looked at the expression of genes that negatively regulate these processes. Tracking Tumor Immunophenotype (http://biocc.hrbmu.edu.cn/TIP/index.jsp) was used to identify immunonegative regulation-related genes (22).

### Tissue Specimens

From September 2021 to March 2022, 10 paired fresh cholangiocarcinoma tissues and neighboring non-tumor tissues were taken from patients who underwent radical surgery at Xi’an Jiaotong University’s First Affiliated Hospital. In addition, between December 2013 and April 2016, 62 patients with cholangiocarcinoma who had a curative procedure were enrolled in a cohort. The protocol was authorized by the First Affiliated Hospital of Xi’an Jiaotong University’s Institutional Medical Ethics Committee (No. XJTU1AF2021LSK-261).

### RNA Isolation and RT-qPCR

The RNeasy Mini Kit (Qiagen) was used to extract total RNA, and the reverse transcription was done with the High Capacity cDNA Reverse Transcription Kit (Thermo Fisher Scientific). A LightCycler480 PCR equipment was used to perform quantitative PCR using SYBR Green dye (Roche). GAPDH was used to standardize the relative fold change in expression.

### Immunohistochemistry (IHC)

Following deparaffin deparaffinization, rehydration and antigen retrieval, the paraffin-embedded slides were incubated with primary anti-PPFIA4 antibody (1:100, Sigma) at 4°C overnight. HRP-conjugated secondary antibody and DAB peroxidase substrate were utilized for immunostaining.

### Statistical Analysis

All statistical analyses were performed using R software (version 4.1.2; https://www.R-project.org). Wilcoxon rank-sum test was used to compare significant differences in continuous variables between the two groups. Pearson correlation analysis was used to analyze the expression correlation between genes. Differentially expressed genes between high and low risk groups were identified based on the R package limma (23) with a differentially expressed gene threshold of |log2FC| > 0.58 and p-values < 0.05. The univariate Cox regression analysis was performed using the R package survival, and multi-factor Cox regression analysis was performed on prognostic model scores to explore the the independent prognostic value of the score. Kaplan-Meier curves were plotted using the R package survminer, and the significance of differences between groups was assessed based on the log-rank test. And p-values < 0.05 were used as the threshold of significance for statistical analysis.

## Results

### Establishment of a Hypoxia-Related Prognostic Model for CHOL

**Figure 1:**
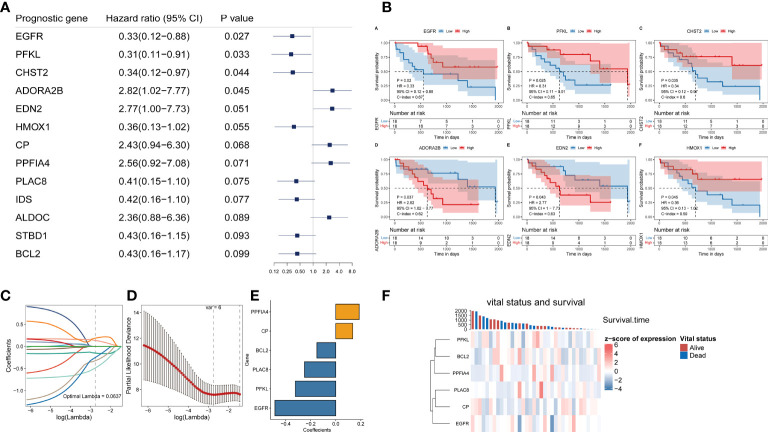
Construction of a hypoxia-related prognostic model for CHOL. **(A)** A forest map showed 13 hypoxia-related signatures identified by univariate Cox proportional hazard regression. **(B)** OS curves of CHOL patients with different PPFIA4 expression. **(C, D)** The LASSO Cox regression model to identify the most robust hypoxia-related signatures. **(E)** Distribution of LASSO coefficients of the hypoxia-related gene signature. **(F)** The heatmap of the vital status, survival and gene expression.

We acquired 200 hypoxia-related genes from the MSigDB database to study the predictive efficiency of hypoxia-related genes in CHOL. EGFR, PFKL, CHST2, ADORA2B, EDN2, HMOX1, CP, PPFIA4, PLAC8, IDS, ALDOC, STBD1, BCL2 were among 13 hypoxia-related genes found to be substantially linked with overall survival (OS) in the TCGA CHOL cohort using univariate Cox hazard analysis (p-value < 0.1, **Figure 1A**). Based on the median value of gene expression (EGFR, PFKL, CHST2, ADORA2B, EDN2, HMOX1), we separated the samples into two groups, and Kaplan-Meier curve analysis revealed that gene expression affected patient survival (log-rank test p-value < 0.05, **Figure 1B**).

Although we initially observed genes with prognostic efficacy in CHOL patients by univariate Cox regression analysis and log-rank test, we further performed Lasso regression analysis based on these 13 genes in order to integrate these informative genes to obtain a more optimal prognostic model, while removing redundant factors and controlling the risk of overfitting. Ten-fold cross-validation was performed under optimal conditions to determine the penalty parameter (λ) of the model. We screened out 6 most predictive factors for OS (**Figures 1C, D**). PPFIA4 and CP were retained as a valid prognostic risk factor, while BCL2, PLAC8, PFKL and EGFR were retained as a prognostic protective factor, Together, they constituted a prognostic risk model associated with hypoxia in CHOL. Based on the linear combination of the expression levels of these genes and the corresponding weights, we could assess the hypoxia-related prognostic risk score for each patient (**Figure 1E**). The corresponding association between the expression of 6 hypoxia-related signature in the prognostic model and the vital status and survival of patients were visualized in **Figure 1F**.

### Independent Validation of the Hypoxia-Related Prognostic Model

**Figure 2:**
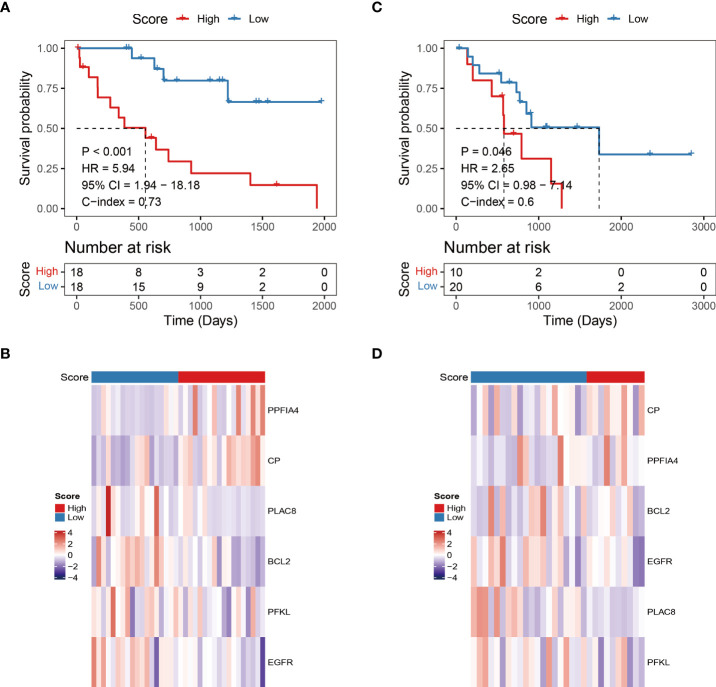
Independent validation of hypoxia-related prognostic models. **(A, C)** Survival curve for low-risk and high-risk subgroups in the training dataset and validation dataset. **(B, D)** The expression heat map of the 6 prognostic hypoxia-related signatures in low-risk and high-risk subgroups training dataset and validation dataset.

The risk score for each patient with CHOL in the TCGA was determined using the hypoxia-related prognostic model for CHOL that was constructed. After obtaining each patient’s risk value, the samples were sorted from low to high in terms of risk score, and scatter plots were used to display the results. The median of all sample risk scores was used as the cutoff, and the samples were divided into two groups: high risk and low risk, the low-risk group’s OS was likewise considerably lower than the high-risk group’s (log-rank test p-value < 0.001, **Figure 2A**). We also calculated heat maps for display to investigate the expression of different genes in the risk model across all genes in different samples, and we discovered that the expression of six hypoxia-related genes in the model differed considerably in the high- and low-risk groups (**Figure 2B**).

To assess the robustness and generalizability of the hypoxia-related prognostic model, we collected CHOL samples from GSE107943 as an independent validation cohort. Patients were divided into high-risk and low-risk groups according to the risk score of the prognostic model. We obtained similar results to the training cohort through an external validation cohort (**Figures 2C, D**).

### Hypoxia-Related Risk Score Is an Independent Prognostic Factor

**Figure 3:**
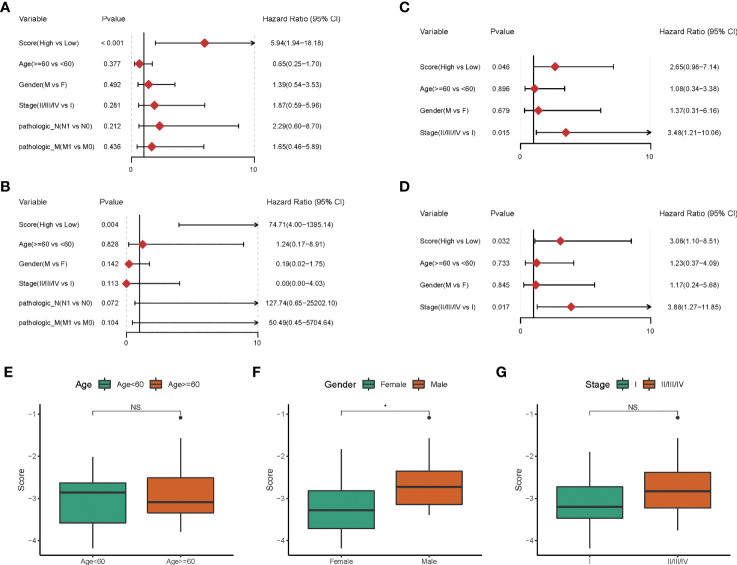
The Hypoxia-related risk score is an independent prognostic factor. **(A-D)** The forest plot of the univariate and multivariate Cox regression analysis shows that the risk score was an independent risk factor for overall survival in training dataset and validation dataset. **(E-G)** The box line chart of the differences in the distribution of risk scores by grouping patients based on their age, gender and stage.

We then explored the independence of the prognostic model in patients with CHOL from TCGA. The univariate Cox regression analysis revealed that the hypoxia-related risk score was the only prognostic factor of OS (HR = 5.94, p-value < 0.001, **Figure 3A**). Further, the multivariate Cox regression analysis confirmed the independent prognostic value of the hypoxia-related risk score (HR = 74.71, p-value = 0.004, **Figure 3B**).

In the GEO cohort, univariate Cox analysis showed that hypoxia-related risk score (HR = 2.65, p-value = 0.046), stage (HR = 3.48, p-value = 0.015) were prognostic factors in patients with CHOL (**Figure 3C**). Hypoxia-related risk score (HR = 3.06, p-value = 0.032) and stage (HR = 3.88, p-value = 0.017) were found to be independent predictive markers in patients with CHOL in a multifactorial Cox analysis (**Figure 3D**), which is consistent with the TCGA cohort’s findings. The prognostic risk scores established in our study might be used as independent risk factors affecting patient prognosis based on the above investigations.

To further corroborate the independence of prognostic risk scores from clinical characteristics, we compared the differences in the distribution of risk scores by grouping patients based on their age, gender and stage, respectively. We found no significant differences in risk scores by age and tumor stage grouping (p-value > 0.05, **Figures 3E, G**), but slightly higher in male patients than in female patients (p-value < 0.05, **Figure 3F**).

### The Interaction Mechanism Between Prognostic Genes

**Figure 4:**
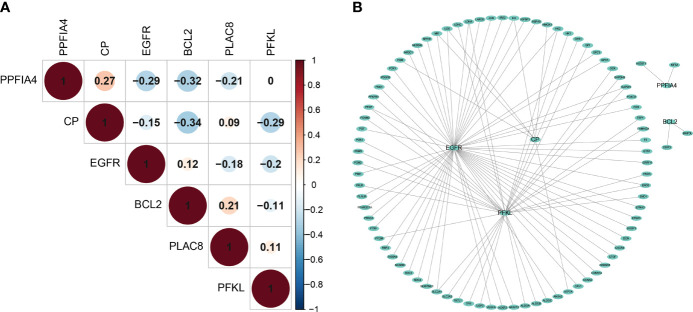
The interaction mechanism between Prognostic Genes. **(A)** The spearman correlation analysis based on the expression of the 6 hypoxia-related signatures. **(B)** The Protein–Protein network interactions including 200 hypoxia-associated genes.

Although the 6 signature genes we obtained from the prognostic model were all involved in the tumor hypoxic state hallmark, but their interactions with other hypoxic genes were not clear, so we examined the co-expression of these genes. Overall, there was no strong co-expression among the hypoxia-related genes involved in the prognostic model (Pearson |r| < 0.34, **Figure 4A**), indicating that the redundant genes with strong associations had been filtered out through LASSO Cox analysis. In addition, we looked at how the hypoxia-related genes in the model interacted with other hypoxia-related genes. We built a PPI network connecting the genes in the prognostic model and other hypoxia-related genes using the interactions of gene-encoded proteins retrieved from the String database online tool (**Figure 4B**).

We found extensive interactions between EGFR, CP, PFKL genes and other hypoxia-associated genes in the model. The model includes hypoxia-related genes, which might play a key role in tumor hypoxia regulation.

### Hypoxia-Related Risk Xcores Reveal Differences in Molecular Tumor Characteristics

**Figure 5:**
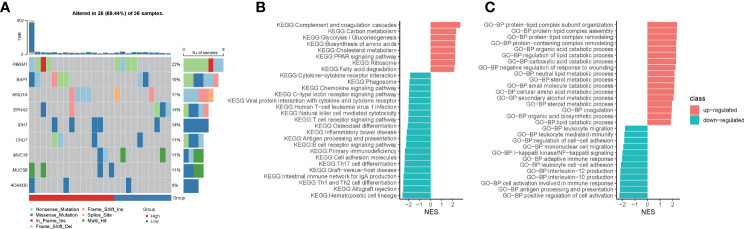
Hypoxia-related risk scores reveal differences in tumor immune microenvironment characteristics. **(A)** The heatmap for mutational gene and mutation type in the high- and low-risk groups. **(B)** The GSEA enrichment analysis for KEGG signaling pathways between the high- and low-risk groups. **(C)** The GSEA enrichment analysis for biological processes from the GO database between the high- and low-risk groups.

To explore the underlying molecular mechanisms of hypoxia-related prognostic models, we first examined somatic mutations in different risk groups in the TCGA-CHOL cohort. Due to the small sample size of the TCGA-CHOL dataset, the comparison of differences in mutation frequencies failed to present significant results (**Figure 5A**, p-value < 0.05). However, we found four patients in the high-risk group enriched for the IDH1 mutation, while only one patient in the low-risk group was enriched for this mutation (**Figure 5A**).

Using GSEA enrichment analysis, we looked into the biological pathways altered by the hypoxia-related predictive risk score in subgroups. Metabolic processes such as “Carbon metabolism”, “Glycolysis/Gluconeogenesis”, “Biosynthesis of amino acids”, “Cholesterol metabolism”, “PPAR signaling pathway” and “Fatty acid degradation” were dramatically increased in the high-risk group (NES > 0, FDR < 0.0001), whereas T cell receptor signaling route, natural killer cell mediated cytotoxicity, antigen processing and presentation, chemokine signaling pathway, and cytokine-cytokine receptor interaction were all significantly downregulated in the high-risk group (NES < 0, FDR < 0.0001, **Figure 5B**). In addition, GSEA enrichment analysis of GO-BP showed that metabolic processes such as “protein-lipid complex subunit organization”, “protein-lipid complex subunit organization”, “protein-lipid complex remodeling”, “protein-containing complex remodeling”, “organic acid catabolic process”, “requlation of lipid catabolic process”, “carboxylic acid catabolic process”, “neutral lipid metabolic process”, “sterol metabolic process”, “small molecule catabolic process”, “cellular amino acid metabolic process”, “secondary alcohol metabolic process”, “steroid metabolic process”, “organic acid biosynthetic process”, “lipid catabolic process” were increased dramatically in the high-risk group (NES > 0, FDR < 0.0001). We observed that “leukocyte migration”, “leukocyte mediated immunity”, “regulation of cell-cell adhesion”, “mononuclear cell migration”, “I-kappaB kinase/NF-kappaB signaling”, “adaptive immune response”, “leukocyte cell-cell adhesion”, “interleukin-12/10 production “, “cell activation involved in immune response”, “antigen processing and presentation”, “positive regulation of cell activation” were significantly downregulated in the high-risk group (NES < 0, FDR < 0.0001; **Figure 5C**). Taken together, we deciphered the tumor molecular signature of the differences between hypoxia-related risk subgroups, suggesting potential molecular mechanisms for the different risk subgroups.

### Hypoxia-Related Risk Scores Reveal Differences in Tumor Immune Microenvironment Characteristics

**Figure 6:**
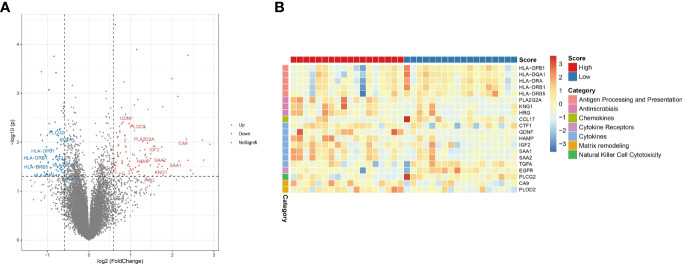
Hypoxia-related risk scores reveal differences in tumor immune microenvironment characteristics. **(A)** The volcano plot of different expression immune-related genes. **(B)** The heatmap of immune-related genes and different immune categories in the high- and low-risk group.

Tumor-immune interactions are crucial in controlling the immune milieu because they incorporate the effects of several regulators. We looked examined how immune regulatory genes were expressed differently in high-risk and low-risk people. We discovered that genes involved in immune modulation were expressed differently in high and low risk groups (**Figure 6A**). We looked examined how divergent genes were expressed in distinct immune regulatory groups.

The genes HLA-DPB1, HLA-DQA1, HLA-DRA, HLA-DRB1 and HLA-DRB5 in the “Antigen Processing and Presentation” category were considerably downregulated in the high-risk group. PLA2G2A, KNG1, HRG, and other genes associated to “antimicrobials” were considerably downregulated in the high-risk group. CCL17, a gene associated to “chemokines,” was considerably downregulated in the high-risk group. The gene EGFR, which belongs to the “Cytokine Receptors” category, was considerably downregulated in the high-risk group. The genes CTF1, GDNF, HAMP, IGF2, SAA1, SAA2, TGFA, which belong to the “cytokines” category, were considerably downregulated in the high-risk group. CA9 and PLOD2, which are connected to “matrix remodeling,” were considerably downregulated in the high-risk group. The gene PLCG2, which is connected to “Natural Killer Cell Cytotoxicity,” was considerably downregulated in the high-risk group. The above results are shown in figures (**Figures 6A, B**). These findings show that immune checkpoint expression differs under the effect of CHOL’s hypoxic condition, affecting the immunological microenvironment’s control.

### The Expression of PPFIA4 Is Dramatically Increased in CHOL Tissues

**Figure 7:**
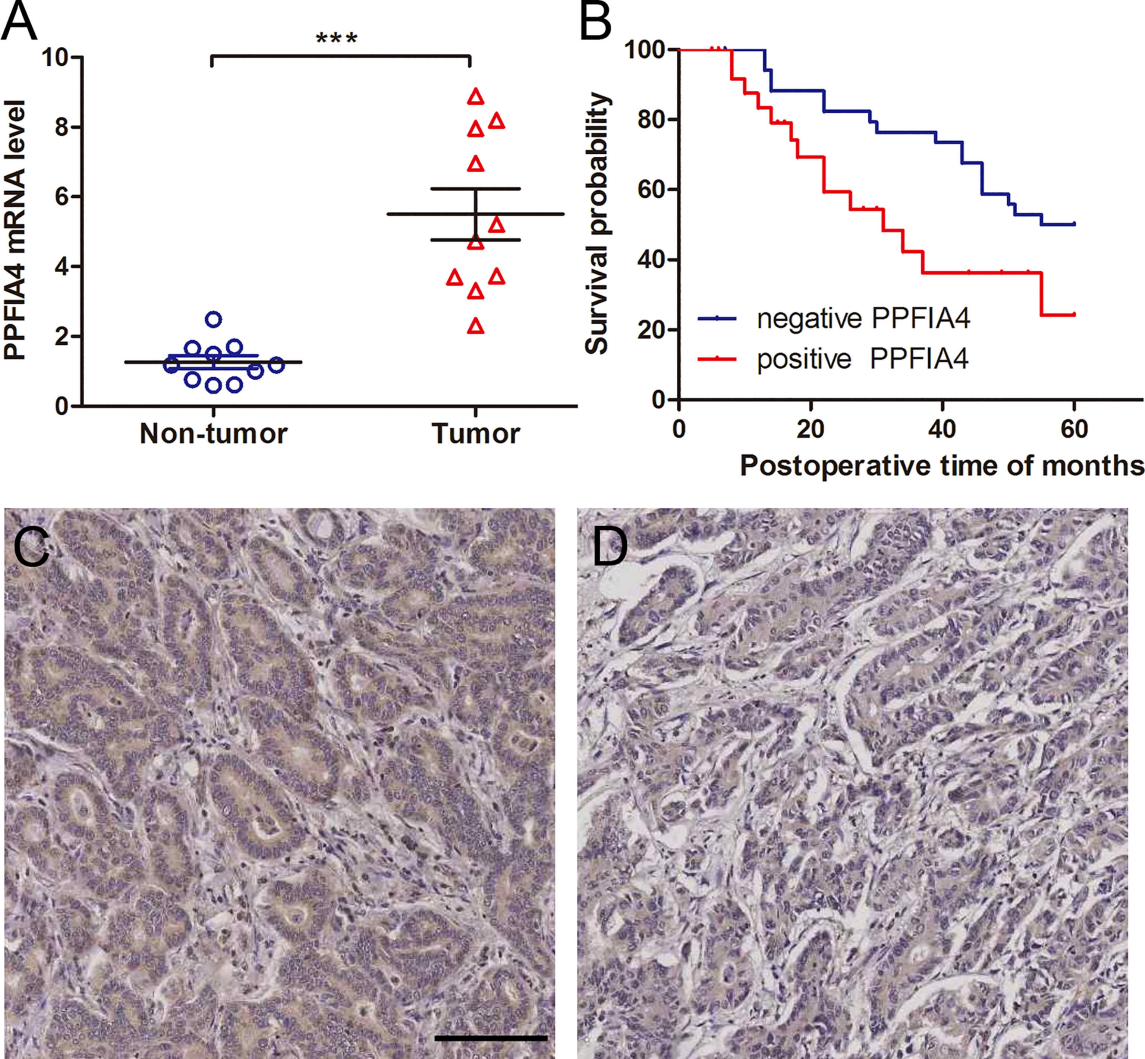
PPFIA4 expression is significantly upregulated in cholangiocarcinoma tissues. **(A)** PPFIA4 mRNA levels were determined by RT-qPCR in 10 pairs of cholangiocarcinoma tissues and matched non-tumor tissues. NT, tumor adjacent non-tumor tissue; T, tumor. ***p-value < 0.001. **(B)** OS curves of cholangiocarcinoma patients with different PPFIA4 expression. **(C)** Representative IHC images of positive PPFIA4 expression. **(D)** Representative IHC images of negative PPFIA4 expression. Scale bar, 100 µm.

To further corroborate these findings, PPFIA4 mRNA levels were measured in 10 matched fresh CHOL tissues with surrounding non-tumor tissues. When compared to non-tumor tissues, the amount of PPFIA4 mRNA in cholangiocarcinoma tissues became much higher (**Figure 7A**). In addition, we investigated PPFIA4 expression in a cohort of patient with cholangiocarcinoma samples from our hospital by IHC assay, which demonstrated predominant expression of PPFIA4 within tumor cells (**Figure 7C, D**). The expression of PPFIA4 was found in 26 of the 62 carcinoma samples (41.9%), whereas the remaining 36 samples (58.1%) were negative. **Table 1** outlined the link between PPFIA4 expression and clinicopathological characteristics. The presence of PPFIA4 was significantly linked to large tumor size, advanced TNM stage, and lymph node metastasis, while there was no correlation of PPFIA4 expression with gender, age, differentiation and tumor stage. Additionally, individuals with high PPFIA4 expression had a poorer 5-year OS prognosis (**Figure 7B**, p-value = 0.03).

**Table 1:** Relationships between PPFIA4 expression and clinicopathological characteristics.

Clinicopathological features	N	PPFIA4 expression		*P*
		Negative	Positive	
Gender		36	26	0.903
Male	40	23	17	
Female	22	13	9	
Age (years)				0.233
< 60	21	10	11	
≥ 60	41	26	15	
Tumor size (cm)				0.014
< 3	34	15	19	
≥ 3	28	21	7	
Differentiation				0.787
Well/moderately	25	14	11	
Poorly	37	22	15	
Tumor stage				0.986
T1-T2	43	25	18	
T3-T4	19	11	8	
Lymph node metastasis				0.020
Absent	39	27	12	
Present	23	9	14	
TNM stage				0.028
I-II	34	24	10	
III-IV	28	12	16	

## Discussion

The molecular mechanisms underlying the development of CHOL are still unclear. It was reported that hypoxia enhances the aggressiveness of CHOL cells (12) and the malignant behavior of tumors can be regulated through hypoxia-related gene (13, 24). Gene signatures have been utilized to predict the prognosis of many malignancies in recent years, and they have proven to be more accurate than TNM staging and histological diagnosis (25, 26). Hypoxia has been identified as a factor impacting patient prognosis that is independent of clinically established prognostic variables such as tumor stage, lymph node status, and tumor grade (27). Although approaches such as nitroimidazole, PET imaging, and IHC biomarker expression have been used to determine the degree of hypoxia in patients’ tumors, the precise proven method remains unknown (28, 29). For a number of malignancies, such as prostate cancer, lung cancer, and breast cancer, prognostic models based on hypoxia-related gene expression have been described (30–32). In this study, we used a Cox and Lasso regression model to analyze 200 hypoxia-related genes, and then screened 6 prognosis-related hypoxia-related genes and constructed a prognostic prediction model for CHOL, and the OS of the high-risk group was significantly lower than that of the low-risk group, and we validated our model using the GEO CHOL date set. The prognostic model we constructed is an ideal model with better sensitivity and specificity.

Phosphofructokinase L (PFKL) includes Binding locations for hypoxia-inducible factor 1 (HIF-1) (33). HIF-1 is a nuclear transcription factor that is active in the oxygen state where the body is adapted to hypoxia or hypoxia. HIF-1α has a certain transcriptional activity and a broad target gene spectrum (34). In hypoxia, the transcriptional and translational levels of HIF-1α can increase exponentially with prolonged hypoxia (35). D-fructose 6-phosphate is converted to D-fructose 1,6-bisphosphonate, a crucial step in glucose metabolism, by an enzyme encoded by PFKL (glycolysis) (36). In the tumor hypoxic region, tumor cells translocate extracellular glucose into the cell *via* glucose transporter proteins on the cell membrane to produce pyruvates and ATP *via* the glycolytic pathway, and LDH-A acts to convert it to lactate (37). The production of lactate constitutes a local acidic environment for tumor cell proliferation and activates the cascade reaction of protein hydrolases to convert pre-matrix metalloproteinases into matrix metalloproteinases, which promote the degradation of the extracellular matrix of cancer cells and facilitate the invasion of surrounding tissues by tumor cells (38). A number of cancers have been identified to have PFKL as a component of the glycolysis process, including lung cancer (39), esophageal cancer (40), neuroblastoma (41) and hepatocellular carcinoma (42). The Bcl-2 protein family is one of the core regulatory mechanisms of apoptosis, which can receive and transmit intrinsic intracellular signals or external environmental stress signals, such as nutrient or hypoxic stress, DNA damage, oncogene over-activation, endoplasmic reticulum stress, etc (43). It was found that Bcl-2-associated transcription factor BCLAF1 was highly expressed under hypoxia with a time-dependent hypoxic effect and positively correlated with HIF1α expression (44). PTPRF Interacting Protein Alpha 4 (PPFIA4) was reported to be glycolysis-associated signature, involved in glycolysis of tumor microenvironment (45). Proliferation and migration of tumor cells in colon cancer are facilitated by PPFIA4’s ability to increase tumor glycolysis (46); PPFIA4 also increases mitochondrial metabolism *via* MTHFD2 in desmoplastic-resistant prostate cancer (47). In our study, we found that PPFIA4 was oncogenic in CHOL and its high expression was associated with poor prognosis of patients. We confirmed the high expression of PPFIA4 in the tumor tissues of CHOL patients through fresh tumor tissues and their corresponding paraneoplastic tissue and previous pathological slides collected clinically. Likewise, we confirmed that high PPFIA4 expression was significantly associated with poor patient prognosis. We hypothesized that the expression status of PPFIA4 in CHOL tumor tissues could reflect the hypoxic status in tumor tissues. It has been discovered that PLAC8 enhances human interstitial extravillous trophoblast cell invasion and migration, and hypoxia dramatically upregulates PLAC8 expression in this cell population (48). In hypoxic conditions, Ceruloplasmin (CP) induces generates oxygen radicals (49), while inducing activation of HIF-1α (50), Overexpression of CP in clear cell renal carcinoma is linked to oncogenic pathways and worse survival rates (51). Epidermal Growth Factor Receptor (EGFR) is a primary regulator of HIF-1 under hypoxic conditions, and mutant EGFR is a prominent regulator of HIF-1a in EGFR mutant NSCLC cells (52). In addition, EGFR was reported that it can blocked anti-cancer miRNA maturation under hypoxic status (53).

Somatic mutations between high- and low-risk groups were compared in this study. Among the high-risk individuals, we observed that Isocitrate Dehydrogenase (NADP(+)) 1 (IDH1) mutations were more common than expected. IDH1 has been linked to tumor hypoxic reprogramming, according to a study (54). Mutations in IDH1 reduce IDH1’s catalytic activity and activate HIF-1 α in glioblastoma cells (55). Wild-type IDH1 suppresses tumor development in renal cell carcinoma *via* degrading HIF-α (56). By using GSEA to examine the variations in cellular activities and pathways between high and low risk groups, we found that metabolic processes and pathways associated with hypoxic state were upregulated, but immune response processes were generally downregulated. Prognostic models developed by our team may be able to respond well to tumor microenvironment hypoxia.

Genes associated to the negative regulation of the tumor immune, such as checkpoints and suppressor-related genes, were examined between high and low risk groups to study the hypoxia/immunity association. Immune checkpoints are targets of immunotherapy, and researchers have found that patients with tumors co-expressing HIF-1α, a subtype of HIF, and PD-L1, an immune checkpoint, have a high risk of tumor recurrence and metastasis as well as lethality (57). In CHOL, hypoxic condition altered the regulatory impact on the immune milieu by modifying the expression of immunological checkpoints. In the high-risk scenario, the hypoxic condition could boost the ability of tumors to escape and invade.

It is possible that our research contained some flaws. In the first place, the research is a retrospective one with a relatively limited sample size. Therefore, the sparse mutation landscape was not suitable for further investigation of genetic factors associated with the hypoxia-related risk score. For example, only three samples showed KRAS and TP53 mutations. In addition, the insufficient clinical information of patients and lack of diversity also hindered the further in-depth analysis. In the present study, most (32/36) of the obtained TCGA-CHOL samples were intrahepatic cholangiocarcinoma, and cholangiocarcinoma at different histological sites may present different molecular features, therefore, study with large sample size would be needed in the future to further analyze cholangiocarcinoma samples from different sites. Nevertheless, the 6-gene hypoxia-related signature identified in our study demonstrated the value to better predict the prognostic status of patients and respond to the hypoxic status of tumor cells.

## Data Availability Statement

The datasets presented in this study can be found in online repositories. The names of the repository/repositories and accession number(s) can be found in the article/supplementary material.

## Ethics Statement

The studies involving human participants were reviewed and approved by First Affiliated Hospital of Xi’an Jiaotong University’s Institutional Medical Ethics Committee. The patients/participants provided their written informed consent to participate in this study.

## Author Contributions

QS, HW, and GW undertook the data analysis and wrote the manuscript. BX and DX helped design the study and edit the manuscript. HW and BX aided in the methodology and data duration. BX and DX assisted in the conception and data representation. All the authors reviewed the data and analysis, read and approved the manuscript.

## Funding

The present study was supported by the National Natural Science Foundation of China (Program No.: 82073271) and Institutional Foundation of The First Affiliated Hospital of Xi’an Jiaotong University (Program No.: 2022YQPY07).

## Conflict of Interest

The authors declare that the research was conducted in the absence of any commercial or financial relationships that could be construed as a potential conflict of interest.

## Publisher’s Note

All claims expressed in this article are solely those of the authors and do not necessarily represent those of their affiliated organizations, or those of the publisher, the editors and the reviewers. Any product that may be evaluated in this article, or claim that may be made by its manufacturer, is not guaranteed or endorsed by the publisher.

## References

[1] Cai Y Cheng N Ye H Li F Song P Tang W. The Current Management of Cholangiocarcinoma: A Comparison of Current Guidelines. Biosci Trends (2016) 10(2):92–102. doi: 10.5582/bst.2016.01048 27026485

[2] Krasinskas AM. Cholangiocarcinoma. Surg Pathol Clin (2018) 11(2):403–29. doi: 10.1016/j.path.2018.02.005 29751883

[3] Tsimafeyeu I Temper M. Cholangiocarcinoma: An Emerging Target for Molecular Therapy. Gastroint Tum (2021) 8(4):153–8. doi: 10.1159/000517258 PMC8546446 34722468

[4] Edge SB Compton CC. The American Joint Committee on Cancer: The 7th Edition of the AJCC Cancer Staging Manual and the Future of TNM. Ann Surg Oncol (2010) 17(6):1471–4. doi: 10.1245/s10434-010-0985-4 20180029

[5] Lee AJ Chun YS. Intrahepatic Cholangiocarcinoma: The AJCC/UICC 8th Edition Updates. Chin Clin Oncol (2018) 7(5):12. doi: 10.21037/cco.2018.07.03 30180751

[6] Hong M Tao S Zhang L Diao LT Huang X Huang S. RNA Sequencing: New Technologies and Applications in Cancer Research. J Hematol Oncol (2020) 13(1):020–01005. doi: 10.1186/s13045-020-01005-x PMC7716291 33276803

[7] Hinshaw DC Shevde LA. The Tumor Microenvironment Innately Modulates Cancer Progression. Cancer Res (2019) 79(18):4557–66. doi: 10.1158/0008-5472.CAN-18-3962 PMC6744958 31350295

[8] Salvatore V Teti G Focaroli S Mazzotti MC Mazzotti A Falconi M. The Tumor Microenvironment Promotes Cancer Progression and Cell Migration. Oncotarget (2017) 8(6):9608–16. doi: 10.18632/oncotarget.14155 PMC5354757 28030810

[9] Petrova V Annicchiarico-Petruzzelli M Melino G Amelio I. The Hypoxic Tumour Microenvironment. Oncogenesis (2018) 7(1):017–0011. doi: 10.1038/s41389-017-0011-9 PMC5833859 29362402

[10] Jing X Yang F Shao C Wei K Xie M Shen H. Role of Hypoxia in Cancer Therapy by Regulating the Tumor Microenvironment. Mol Canc (2019) 18(1):019–1089. doi: 10.1186/s12943-019-1089-9 PMC6844052 31711497

[11] Muz B de la Puente P Azab F Azab AK. The Role of Hypoxia in Cancer Progression, Angiogenesis, Metastasis, and Resistance to Therapy. Hypoxia (2015) 3:83–92. doi: 10.2147/HP.S93413 27774485 PMC5045092

[12] Seubwai W Kraiklang R Wongkham C Wongkham S. Hypoxia Enhances Aggressiveness of Cholangiocarcinoma Cells. Asian Pac J Cancer Prev (2012) 13:53–8. 23480765

[13] Hou P Kang Y Luo J. Hypoxia-Mediated miR-212-3p Downregulation Enhances Progression of Intrahepatic Cholangiocarcinoma Through Upregulation of Rab1a. Cancer Biol Ther (2018) 19(11):984–93. doi: 10.1080/15384047.2018.1456608 PMC6301827 29672195

[14] Bhuria V Xing J Scholta T Bui KC Nguyen MLT Malek NP. Hypoxia Induced Sonic Hedgehog Signaling Regulates Cancer Stemness, Epithelial-To-Mesenchymal Transition and Invasion in Cholangiocarcinoma. Exp Cell Res (2019) 385(2):18. doi: 10.1016/j.yexcr.2019.111671 31634481

[15] Colaprico A Silva TC Olsen C Garofano L Cava C Garolini D. TCGAbiolinks: An R/Bioconductor Package for Integrative Analysis of TCGA Data. Nucleic Acids Res (2016) 44(8):23. doi: 10.1093/nar/gkv1507 PMC4856967 26704973

[16] Engebretsen S Bohlin J. Statistical Predictions With Glmnet. Clin Epigenet (2019) 11(1):123. doi: 10.1186/s13148-019-0730-1 PMC6708235 31443682

[17] Wang S Su W Zhong C Yang T Chen W Chen G. An Eight-CircRNA Assessment Model for Predicting Biochemical Recurrence in Prostate Cancer. Front Cell Dev Biol (2020) 8(599494). doi: 10.3389/fcell.2020.599494 PMC7758402 33363156

[18] Tilford CA Siemers NO. Gene Set Enrichment Analysis. Methods Mol Biol (2009) 563:99–121. doi: 10.1007/978-1-60761-175-2_6 19597782

[19] Yu G Wang LG Han Y He QY. Clusterprofiler: An R Package for Comparing Biological Themes Among Gene Clusters. Omics (2012) 16(5):284–7. doi: 10.1089/omi.2011.0118 PMC3339379 22455463

[20] Mayakonda A Lin DC Assenov Y Plass C Koeffler HP. Maftools: Efficient and Comprehensive Analysis of Somatic Variants in Cancer. Genome Res (2018) 28(11):1747–56. doi: 10.1101/gr.239244.118 PMC6211645 30341162

[21] Chen DS Mellman I. Oncology Meets Immunology: The Cancer-Immunity Cycle. Immunity (2013) 39(1):1–10. doi: 10.1016/j.immuni.2013.07.012 23890059

[22] Xu L Deng C Pang B Zhang X Liu W Liao G. TIP: A Web Server for Resolving Tumor Immunophenotype Profiling. Cancer Res (2018) 78(23):6575–80. doi: 10.1158/0008-5472.CAN-18-0689 30154154

[23] Ritchie ME Phipson B Wu D Hu Y Law CW Shi W. Limma Powers Differential Expression Analyses for RNA-Sequencing and Microarray Studies. Nucleic Acids Res (2015) 43(7):20. doi: 10.1093/nar/gkv007 PMC4402510 25605792

[24] Huang MP Gu SZ Huang B Li GW Xiong ZP Tang T. Apatinib Inhibits Angiogenesis in Intrahepatic Cholangiocarcinoma by Regulating the Vascular Endothelial Growth Factor Receptor-2/Signal Transducer and Activator of Transcription Factor 3/Hypoxia Inducible Factor 1 Subunit Alpha Signaling Axis. Pharmacology (2021) 106(9-10):509–19. doi: 10.1159/000514410 34412054

[25] Chlis NK Bei ES Zervakis M. Introducing a Stable Bootstrap Validation Framework for Reliable Genomic Signature Extraction. IEEE/ACM Trans Comput Biol Bioinform (2018) 15(1):181–90. doi: 10.1109/TCBB.2016.2633267 27913357

[26] Karamichalis R Kari L Konstantinidis S Kopecki S Solis-Reyes S. Additive Methods for Genomic Signatures. BMC Bioinf (2016) 17(1):016–1157. doi: 10.1186/s12859-016-1157-8 PMC4994249 27549194

[27] Jubb AM Buffa FM Harris AL. Assessment of Tumour Hypoxia for Prediction of Response to Therapy and Cancer Prognosis. J Cell Mol Med (2010) 14(1-2):18–29. doi: 10.1111/j.1582-4934.2009.00944.x 19840191 PMC3837600

[28] Koch CJ Evans SM. Optimizing Hypoxia Detection and Treatment Strategies. Semin Nucl Med (2015) 45(2):163–76. doi: 10.1053/j.semnuclmed.2014.10.004 PMC4365940 25704388

[29] Walsh JC Lebedev A Aten E Madsen K Marciano L Kolb HC. The Clinical Importance of Assessing Tumor Hypoxia: Relationship of Tumor Hypoxia to Prognosis and Therapeutic Opportunities. Antiox Redox Signal (2014) 21(10):1516–54. doi: 10.1089/ars.2013.5378 PMC4159937 24512032

[30] Yang L Roberts D Takhar M Erho N Bibby BAS Thiruthaneeswaran N. Development and Validation of a 28-Gene Hypoxia-Related Prognostic Signature for Localized Prostate Cancer. EBioMedicine (2018) 31:182–9. doi: 10.1016/j.ebiom.2018.04.019 PMC6014579 29729848

[31] Lane B Khan MT Choudhury A Salem A West CML. Development and Validation of a Hypoxia-Associated Signature for Lung Adenocarcinoma. Sci Rep (2022) 12(1):022–05385. doi: 10.1038/s41598-022-05385-7 PMC8789914 35079065

[32] Wang J Wang Y Xing P Liu Q Zhang C Sui Y. Development and Validation of a Hypoxia-Related Prognostic Signature for Breast Cancer. Oncol Lett (2020) 20(2):1906–14. doi: 10.3892/ol.2020.11733 PMC7377061 32724434

[33] Semenza GL Roth PH Fang HM Wang GL. Transcriptional Regulation of Genes Encoding Glycolytic Enzymes by Hypoxia-Inducible Factor 1. J Biol Chem (1994) 269(38):23757–63. doi: 10.1016/S0021-9258(17)31580-6 8089148

[34] Qiu MZ Han B Luo HY Zhou ZW Wang ZQ Wang FH. Expressions of Hypoxia-Inducible Factor-1α and Hexokinase-II in Gastric Adenocarcinoma: The Impact on Prognosis and Correlation to Clinicopathologic Features. Tum Biol (2011) 32(1):159–66. doi: 10.1007/s13277-010-0109-6 20845004

[35] Liu W Shen SM Zhao XY Chen GQ. Targeted Genes and Interacting Proteins of Hypoxia Inducible Factor-1. Int J Biochem Mol Biol (2012) 3(2):165–78. PMC3388736 22773957

[36] Yi W Clark PM Mason DE Keenan MC Hill C Goddard WA 3rd. Phosphofructokinase 1 Glycosylation Regulates Cell Growth and Metabolism. Sci (New York NY). (2012) 337(6097):975–80. doi: 10.1126/science.1222278 PMC3534962 22923583

[37] Kianercy A Veltri R Pienta KJ. Critical Transitions in a Game Theoretic Model of Tumour Metabolism. Interface Foc (2014) 4(4):20140014. doi: 10.1098/rsfs.2014.0014 PMC4071509 25097747

[38] Vlachostergios PJ Oikonomou KG Gibilaro E Apergis G. Elevated Lactic Acid is a Negative Prognostic Factor in Metastatic Lung Cancer. Cancer Biomark: Sec A Dis Mark (2015) 15(6):725–34. doi: 10.3233/CBM-150514 PMC12965472 26406401

[39] Yang J Li J Le Y Zhou C Zhang S Gong Z. PFKL/miR-128 Axis Regulates Glycolysis by Inhibiting AKT Phosphorylation and Predicts Poor Survival in Lung Cancer. Am J Cancer Res (2016) 6(2):473–85. PMC4859674 27186417

[40] Zheng C Yu X Liang Y Zhu Y He Y Liao L. Targeting PFKL With Penfluridol Inhibits Glycolysis and Suppresses Esophageal Cancer Tumorigenesis in an AMPK/FOXO3a/BIM-Dependent Manner. Acta Pharm Sin B (2022) 12(3):1271–87. doi: 10.1016/j.apsb.2021.09.007 PMC9069409 35530161

[41] Zhang S Hua Z Ba G Xu N Miao J Zhao G. Antitumor Effects of the Small Molecule DMAMCL in Neuroblastoma *via* Suppressing Aerobic Glycolysis and Targeting PFKL. Cancer Cell Int (2021) 21(1):619. doi: 10.1186/s12935-021-02330-y 34819091 PMC8613996

[42] Feng Y Zhang Y Cai Y Liu R Lu M Li T. A20 Targets PFKL and Glycolysis to Inhibit the Progression of Hepatocellular Carcinoma. Cell Death Dis (2020) 11(2):89. doi: 10.1038/s41419-020-2278-6 32015333 PMC6997366

[43] Bruckheimer EM Cho SH Sarkiss M Herrmann J McDonnell TJ. The Bcl-2 Gene Family and Apoptosis. Adv Biochem Engineering/biotechnol (1998) 62:75–105. doi: 10.1007/BFb0102306 9755641

[44] Wen Y Zhou X Lu M He M Tian Y Liu L. Bclaf1 Promotes Angiogenesis by Regulating HIF-1α Transcription in Hepatocellular Carcinoma. Oncogene (2019) 38(11):1845–59. doi: 10.1038/s41388-018-0552-1 PMC6462866 30367150

[45] Xu F Xu H Li Z Huang Y Huang X Li Y. Glycolysis-Based Genes Are Potential Biomarkers in Thyroid Cancer. Front Oncol (2021) 11:534838. doi: 10.3389/fonc.2021.534838 33981593 PMC8107473

[46] Huang J Yang M Liu Z Li X Wang J Fu N. PPFIA4 Promotes Colon Cancer Cell Proliferation and Migration by Enhancing Tumor Glycolysis. Front Oncol (2021) 11:653200. doi: 10.3389/fonc.2021.653200 34094943 PMC8173052

[47] Zhao R Feng T Gao L Sun F Zhou Q Wang X. PPFIA4 Promotes Castration-Resistant Prostate Cancer by Enhancing Mitochondrial Metabolism Through Mthfd2. J Exp Clin Cancer Res: CR. (2022) 41(1):125. doi: 10.1186/s13046-022-02331-3 35382861 PMC8985307

[48] Chang WL Liu YW Dang YL Jiang XX Xu H Huang X. PLAC8, a New Marker for Human Interstitial Extravillous Trophoblast Cells, Promotes Their Invasion and Migration. Dev (Cambridge England) (2018) 145(2). doi: 10.1242/dev.148932 PMC5825838 29361555

[49] Chepelev NL Willmore WG. Regulation of Iron Pathways in Response to Hypoxia. Free Radical Biol Med (2011) 50(6):645–66. doi: 10.1016/j.freeradbiomed.2010.12.023 21185934

[50] Martin F Linden T Katschinski DM Oehme F Flamme I Mukhopadhyay CK. Copper-Dependent Activation of Hypoxia-Inducible Factor (HIF)-1: Implications for Ceruloplasmin Regulation. Blood (2005) 105(12):4613–9. doi: 10.1182/blood-2004-10-3980 15741220

[51] Zhang Y Chen Z Chen JG Chen XF Gu DH Liu ZM. Ceruloplasmin Overexpression is Associated With Oncogenic Pathways and Poorer Survival Rates in Clear-Cell Renal Cell Carcinoma. FEBS Open bio. (2021) 11(11):2988–3004. doi: 10.1002/2211-5463.13283 PMC8564342 34449964

[52] Le X Nilsson M Goldman J Reck M Nakagawa K Kato T. Dual EGFR-VEGF Pathway Inhibition: A Promising Strategy for Patients With EGFR-Mutant NSCLC. J Thorac Oncol (2021) 16(2):205–15. doi: 10.1016/j.jtho.2020.10.006 33096270

[53] Shen J Xia W Khotskaya YB Huo L Nakanishi K Lim SO. EGFR Modulates microRNA Maturation in Response to Hypoxia Through Phosphorylation of AGO2. Nature (2013) 497(7449):383–7. doi: 10.1038/nature12080 PMC3717558 23636329

[54] Chang S Yim S Park H. The Cancer Driver Genes IDH1/2, JARID1C/ KDM5C, and UTX/ KDM6A: Crosstalk Between Histone Demethylation and Hypoxic Reprogramming in Cancer Metabolism. Exp Mol Med (2019) 51(6):1–17. doi: 10.1038/s12276-019-0230-6 PMC6586683 31221981

[55] Zhao S Lin Y Xu W Jiang W Zha Z Wang P. Glioma-Derived Mutations in IDH1 Dominantly Inhibit IDH1 Catalytic Activity and Induce HIF-1alpha. Sci (New York NY). (2009) 324(5924):261–5. doi: 10.1126/science.1170944 PMC3251015 19359588

[56] Chen S Wang Y Xiong Y Peng T Lu M Zhang L. Wild-Type IDH1 Inhibits the Tumor Growth Through Degrading HIF-α in Renal Cell Carcinoma. Int J Biol Sci (2021) 17(5):1250–62. doi: 10.7150/ijbs.54401 PMC8040470 33867843

[57] Leone RD Emens LA. Targeting Adenosine for Cancer Immunotherapy. J Immunother Cancer. (2018) 6(1):57. doi: 10.1186/s40425-018-0360-8 29914571 PMC6006764

